# MEASURING THE EFFECTS OF A CYCLING PROGRAM ON QUALITY OF LIFE OF OLDER ADULTS LIVING IN LONG TERM CARE

**DOI:** 10.1093/geroni/igz038.3123

**Published:** 2019-11-08

**Authors:** Victoria Cotnam, Aleksandra Zecevic

**Affiliations:** Western University, London, Ontario, Canada

## Abstract

Cycling Without Age (CWA) is a program offered in long-term care (LTC) homes around the world that allows older adults who are unable to ride a bicycle the pleasure of a bike ride again. Two residents sit in the front bench seat of a trishaw, and a volunteer bike pilot pedals the bike. A variety of anecdotal benefits have been reported and no study has rigorously measured the effects of this program. The purpose of this research is to measure the effects of the CWA program on happiness and quality of life of LTC home residents, through observation of an existing program in a Canadian LTC home. A total of 24 residents were purposefully recruited in a biking group (n=23) who were biked twice a week for 12 weeks, and a strolls group (n=16) who went for outdoors walks or wheelchair rides for the same period of time. Data on pain, cognition, social engagement, and aggressive behaviour was harvested from the Resident-Assessment Instrument – Minimum Data Set (RAI-MDS). Happiness was measured pre and post all bike rides and strolls using a visual analogue scale, and the LTC QoL assessment was used to assess QOL. Findings show that biking group scored higher on happiness after bike rides compared to before, as well as compared to strolls. Bike group QOL scores are higher at the end of the 12 weeks than were strolls group. In summary, CWA shows potential to increase QOL and happiness of residents living in LTC.

